# CO_2_ Separation and Capture Properties of Porous Carbonaceous Materials from Leather Residues

**DOI:** 10.3390/ma6104641

**Published:** 2013-10-18

**Authors:** José M. Bermúdez, Pablo Haro Dominguez, Ana Arenillas, Jaume Cot, Jens Weber, Rafael Luque

**Affiliations:** 1 Departamento de Química Orgánica, Universidad de Córdoba, Edif. Marie Curie, Ctra Nnal IV-A, Km 396, Córdoba E14014, Spain; E-Mail: jmbermudez@incar.csic.es; 2 Instituto Nacional del Carbón, CSIC, Apartado 73, Oviedo 33080, Spain; E-Mail: aapuente@incar.csic.es; 3 Max Planck Institute for Colloids and Interfaces, Department of Colloid Chemistry, Science Park Golm, Potsdam 14424, Germany; E-Mails: pablo.harodominguez@mpikg.mpg.de (P.H.D.); jens.weber@mpikg.mpg.de (J.W.); 4 Institut de Quiımica Avancada de Catalunya (IQAC)—Consejo Superior de Investigaciones Cientıficas (CSIC), C/Jordi Girona, 18–26, Barcelona 08034, Spain; E-Mail: jaume.cot@iqac.csic.es

**Keywords:** CO_2_ sequestration, leather residues, carbon materials, porous materials

## Abstract

Carbonaceous porous materials derived from leather skin residues have been found to have excellent CO_2_ adsorption properties, with interestingly high gas selectivities for CO_2_ (α > 200 at a gas composition of 15% CO_2_/85% N_2_, 273K, 1 bar) and capacities (>2 mmol·g^−1^ at 273 K). Both CO_2_ isotherms and the high heat of adsorption pointed to the presence of strong binding sites for CO_2_ which may be correlated with both: N content in the leather residues and ultrasmall pore sizes.

## 1. Introduction

The combination of more environmentally friendly and low impact technologies in manufacturing processes that provide improved efficiencies as well as waste minimization have been highlighted to be critical assets to favor industrial development for the benefit of future societies in a more sustainable manner [1]. In this regard, greener processes and technologies aiming to valorize residues to valuable products (e.g., materials, chemicals and energy) should be the focus of future research endeavors in our aim to meet sustainability targets and to face the lack of resources we will experience in coming decades [2]. Waste valorization, differently understood from basic management of residues, can offer important advantages to enhance the value of residues via greener processing to valuable products, both from the environmental and economic viewpoints [2].

Leather industries generate exceptional quantities of residues which have been rather under utilized to date [3,4]. Managing such waste is essential. The majority of this waste (wet residues, >650,000 tons per year generated worldwide) [5], which contains metals and chemicals including chromium, titanium and others, currently has no uses different from landfilling. More importantly, some of those residues including unprocessed defective skin, processed leather bits and related others produced in large scale (dry waste, accounting for over 150,000 tons per year worldwide) [5], constitute a major issue to leather processing companies that do not generally find alternatives to manage them. Fully or at least partially processed leather skin waste can in principle be a potentially promising feedstock to valorize to a range of valuable products with interesting properties.

Spain has an important leather industry both at the North (Catalonia) and South (Andalucia) from which we have been extensively collaborating in past years [6,7]. Along these lines, we recently developed a proof of concept methodology to valorize leather skin residues (processed and unprocessed) to potentially interesting materials for a series of applications [6,7,8]. Other literature reports have also focused on the conversion of bio-collagenic residues into carbonaceous materials by means of a facile carbonization approach at high temperatures (>900 °C) as alternative methodology to existing chemical vapor deposition methods [8]. The carbonaceous-derived materials were found to have a partially graphitized structure with onion like morphology containing nitrogen and oxygen into their composition, resulting in multifunctional properties with potential applications in battery electrodes [9].

Inspired by such previous studies, particularly related to the high N content of leather residues (contained in proteins ubiquitous in animal skin), we were interested in the conversion of leather skin residues into greener porous carbonaceous materials suitable for adsorption applications. N-containing materials have been reported to have interesting adsorption properties in various applications, particularly for CO_2_ sequestration [10,11,12].

In this work, we present a preliminary assessment of the potential of carbonaceous materials from leather-derived residues on CO_2_ adsorption. We demonstrate that such materials obtained from (un)processed leather skin can have a very promising CO_2_ adsorption with an interesting gas selectivity over N_2_.

## 2. Results and Discussion

**Figure 1:**
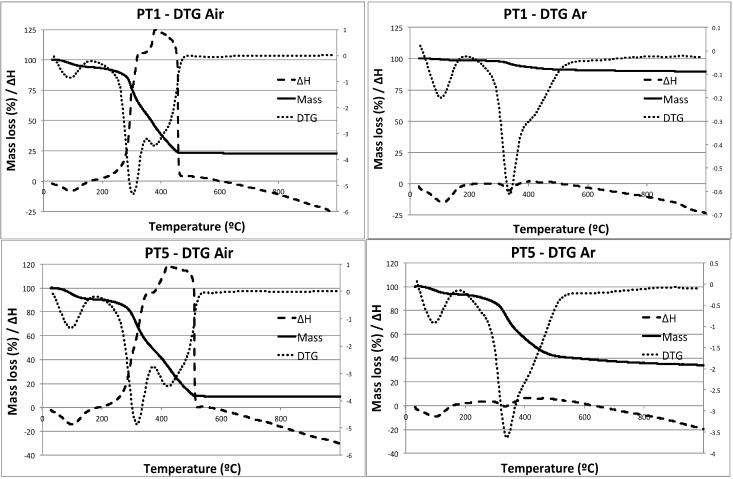
Thermal gravimetric and differential thermal analysis (TG/DTA) profiles of PT1 and PT5 under air (left plot) and argon (right plot).

Thermal gravimetric and differential thermal analysis (TG/DTA) measurements were initially conducted to carefully plan the selection of the carbonization temperature for the materials. Figure 1 clearly depicts the remarkable differences between carbonized materials under air and argon. Leather residues carbonized under air showed the typical small mass loss associated to physisorbed water (*ca.* 100–110 °C, <10% mass loss) which was followed by a highly exothermic band between 300 and 480 °C, corresponding to the decomposition and burning of the residue into CO_2_ and water as well as some NO*_x_* species [in good agreement with TG/MS (thermogravimetry mass spectrometry) results, not shown], with a mass loss of over 70% (Figure 1). A mass reduction of *ca.* 75%–90% was observed in all samples, regardless of the starting material. Comparatively, the carbonized materials under inert atmosphere (Ar or Nitrogen) exhibited a remarkably different profile in which no significant mass loss (less than 50%) was observed in the worst case scenario. Similar mass loss steps were observed in the case of carbonization under inert atmosphere but to a lower extend. These were typically around 5–20 wt %. From TG/DTA curves we could infer two key points to select the temperatures for carbonization: prior to the important mass loss in the systems (around 350 °C) and close after the significant decomposition step of samples (600 °C, to ensure a full sample carbonization). Temperatures higher than 600 °C were ruled out as in principle they were believed to not offer very different information to results obtained at 600 °C and obviously required of a larger energy input in the carbonization process.

Materials carbonized under inert atmosphere (e.g., Ar and N_2_) were the focus of subsequent studies included in this work.

**Figure 2:**
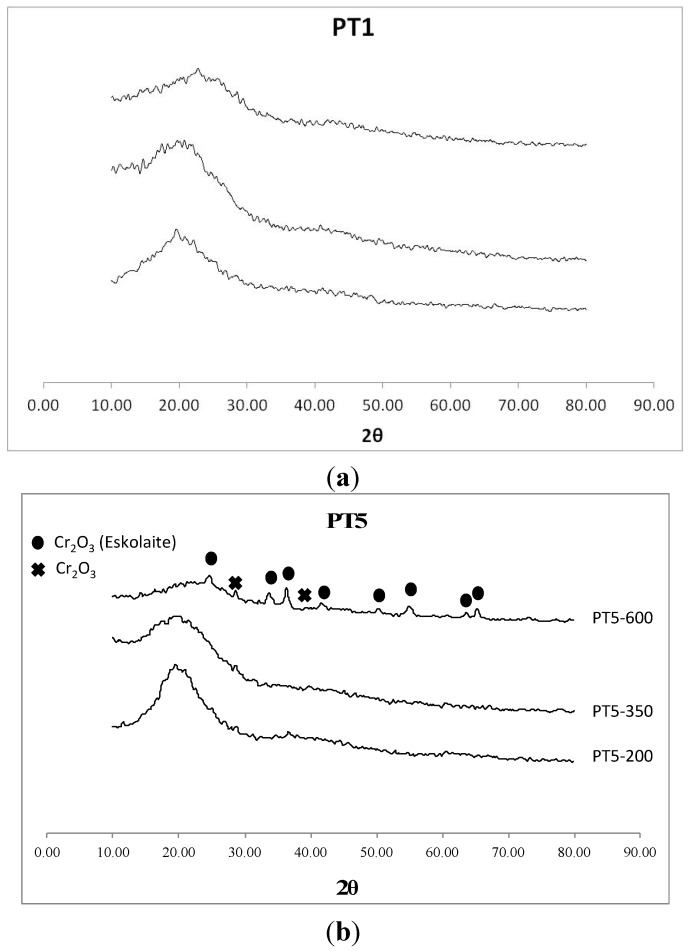
X-Ray diffraction (XRD) patterns of PT1 and PT5 carbonized under nitrogen at 200, 350 and 600 °C.

X-ray Diffraction (XRD) (patterns of carbonaceous materials obtained from processed leather residues (Figure 2, see also supporting information) pointed out the existence of an amorphous phase corresponding to a carbonaceous material (broad band at *ca.* 2θ = 20°, Figure 2a) which gradually decreases with temperature to give rise to several crystalline peaks corresponding to the various species present in the leather skin residue depending on their origin and treatment/processing (Figure 2b, supporting information). In most cases, processed skins exhibited diffraction lines corresponding to various oxide phases of chromium (eskolaite, Cr_2_O_3_) and titanium (rutile and anatase, TiO_2_) (Figure 2, see also supporting information). Cr and Ti contents in the materials were measured by inductively coupled plasma mass spectrometry (ICP/MS), showing <5 wt % Cr (for PT4 and PT5) and Ti (*ca.* 4.6 wt % for PT2) in the investigated materials.

Diffuse reflectance infrared Fourier transform (DRIFTS) spectra recorded for selected samples (not shown, see supporting information) proved the transformation of the parent leather skin residues into carbonaceous materials. In this sense, several bands of the parent leather materials, including O–H, C–N (from proteins) and C–C bands are mostly lost in the carbonaceous materials at higher temperatures (600 °C), leading to an amorphous carbonaceous type of structure with bands in the 900–1000 cm^−1^ corresponding to aromatic groups for most materials (see supporting information).

Nitrogen adsorption isotherms collected at 77.4 K do not show any signs of microporosity, with profiles typical of low porosity materials (see also supporting information). The steep increase observed at high relative pressure is typically associated with either a macroporous structure or agglomerated small particles. Such morphology is in accordance with transmission electron microscopy (TEM) investigations (see below). In either case, this can be beneficial for gas separation applications facilitating a fast mass transfer to adsorption sites as later discussed. The absence of any measurable microporosity by classic N_2_ adsorption at 77.4 K (at first sight contradictory to the high CO_2_ capacity observed in the materials) is however well known from both carbon [13,14,15,16] and polymer research [17]. It points to the presence of ultrasmall pores, which cannot be easily filled by Nitrogen at 77.4 K either due to size or kinetic restrictions. Table 1 summarizes the specific surface areas and pore volumes of the carbonized material.

**Figure 3:**
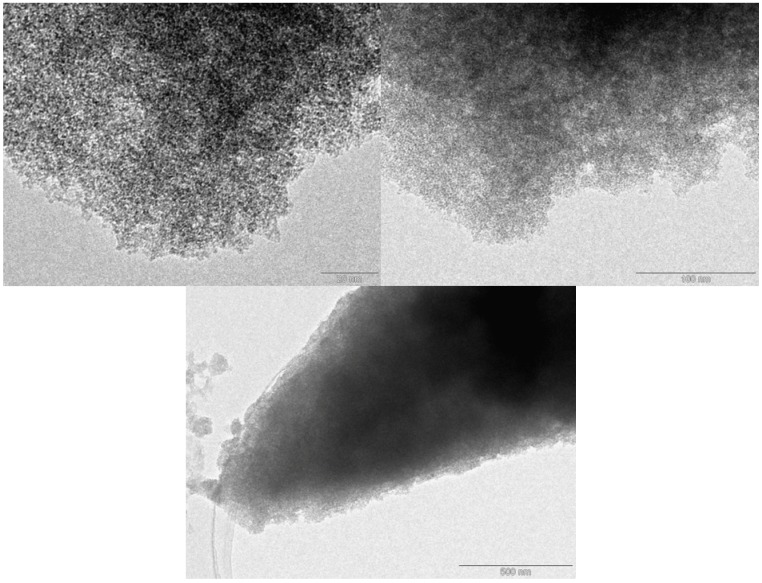
Transmission electron microscopy (TEM) micrographs of PT1-600 materials.

The presence of a distinct morphology of small particulates and/or developed interparticular macroporosity was also evidenced in TEM micrographs (Figure 3). Not much more information can be obtained from these images that otherwise clearly shows amorphous-like materials that resemble those of typical carbons [18].

Upon characterization, the CO_2_ adsorption capacity of prepared carbonaceous materials was investigated. These studies were based on previous reports that suggested that N-containing materials possess improved CO_2_ adsorption properties as compared to pristine porous carbons based on various binding modes of CO_2_ to Nitrogen [15,16,19,20,21,22,23,24]. The deeper reasons of the impact of N-doping on the CO_2_ uptake properties are still a matter of discussion. Hydrogen bonding interactions between C–H and N–H and CO_2_ were recently suggested to be one of the main reasons for such affinity by Qiao *et al.* [19]. These results challenged the extensively reported argument that related improved CO_2_ adsorption on N doped carbons as a consequence of acid-base interactions between N functionalities and CO_2_ [25]. More recent reports do, however, suggest that the actual micropore size may be the main reason for the observation of high CO_2_ uptake [15,16,26]. This can be understood from the fact that CO_2_ fills very narrow pores by volume-filling, while wider pores (*W* > 1.2 nm) are only surface covered within the commonly analyzed pressure range (*T*: 0–25 °C, *P*: 0–1 bar). However, we doubt that polar functionalities at the surface can lead to additionally enhanced adsorption tendency.

**Table 1:** Porosity properties of carbonized materials investigated in this work.

Material	S_BET_ (m^2^/g)	Pore Volume (cm^3^/g)
PT1*	–	–
PT1-350*	< 10	0.09
PT1-600*	29	0.10
PT2*	–	–
PT2-350*	< 10	0.06
PT2-600*	21	0.07
PT3*	–	–
PT3-350*	< 5	–
PT3-600*	< 5	–
PT4*	–	–
PT4-350*	< 5	–
PT4-600*	–	–
PT5-350*	< 5	–
PT5-600*	10	0.05

* A detailed explanation of the nomenclature used can be found in the Experimental Section.

**Figure 4:**
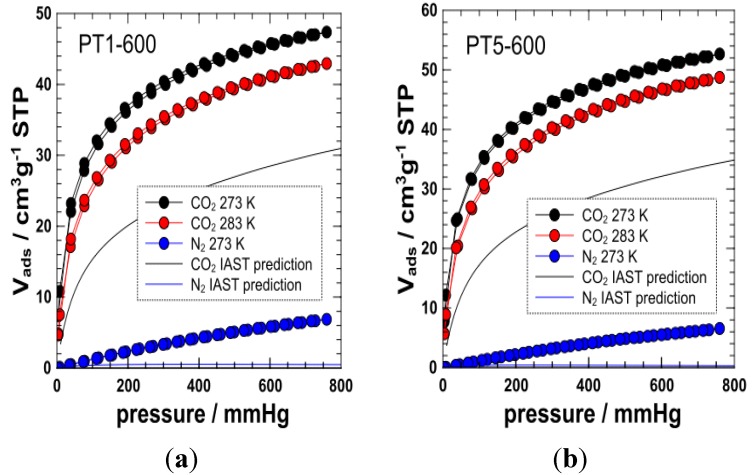
Symbols: Experimental adsorption/desorption isotherms of CO_2_ (273 K, 283K) and N_2_ (273K) of PT1-600 (left plot) and PT5-600 (**a**); Lines: predicted adsorption isotherms of CO_2_ and N_2_ at 273K of PT1-600 (**b**) and PT5-600 (right plot) as determined from ideal adsorbed solution theory (IAST) modeling (gas composition: 0.15/0.85 CO_2_/N_2_).

CO_2_ adsorption properties of selected materials carbonized under nitrogen (PT1-600 and PT5-600) were analyzed in more detail. Initial adsorption experiments were undertaken at 273.15 K (Figure 4), which allows a first screening of the porosity and the extraction of the pore size distribution and specific surface area using a GCMC (grand canonical monte carlo) methodology [27]. Materials PT1-600 and PT5-600 showed a significant CO_2_ uptake of 2.11 mmol·g^−1^ (PT1-600) and 2.35 mmol·g^−1^ (PT5-600) and were consequently analyzed with regard to their structural characteristics, heat of adsorption and adsorption selectivity. At this stage, the adsorption of a conventionally carbonized material (under air) was also compared to such nitrogen atmosphere carbonized materials (ESI). Interestingly, their analogues carbonized under air exhibited a very low CO_2_ uptake accounting for an almost negligible <0.1 mmol·g^−1^. The observed partial removal of nitrogen under the calcination step under air (see exothermic band in the 300–500 °C range from TG/DTA, Figure 1 and ESI) is believed to be one of the reasons accounting for the poor CO_2_ uptake in these samples as well as the potential destruction of the ultramicroporous framework by oxidation processes.

Translating the isotherms of nitrogen-carbonized materials into a pore size distribution (PSD) and specific surface area applying the above-mentioned GCMC methodology (see ESI), a very narrow PSDs could be found, centered at pore widths of ~0.5 nm. Overall, both samples do show a very similar PSD and the vast majority of their pores have widths < 0.7 nm. Such small pore sizes can explain the absence of N_2_ uptake at 77.4 K. Specific surface areas calculated from the GCMC method were found to be 452 m^2^·g^−1^ (PT1-600) and 475 m^2^·g^−1^ (PT5-600), respectively.

**Figure 5:**
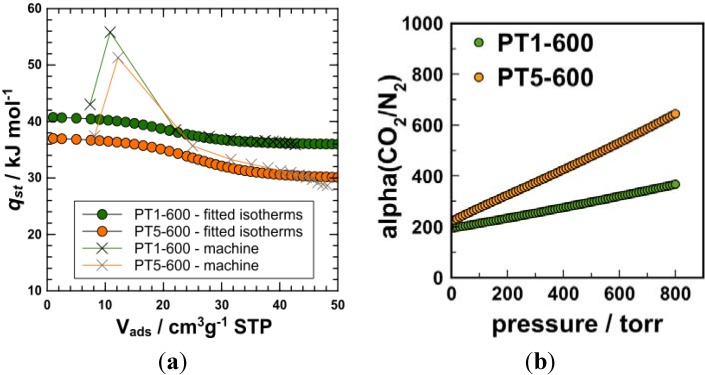
Isosteric heat of carbon dioxide adsorption of (**a**) PT1-600 and PT5-600; and (**b**) IAST prediction of the gas selectivity of PT1-600 and PT5-600 for the gas pair CO_2_/N_2_ 0.15/0.85 composition at 273 K.

CO_2_ adsorption at 283 K was subsequently measured in order to access the isosteric heat of adsorption *q_st_*. Calculation was based on the routine provided by the software package of the gas adsorption instrument manufacturer and on a routine suggested by Krishna, Long and coworkers (Figure 5) [28]. Interestingly, the software did overestimate the heat of adsorption in the low-coverage region, probably, due to bad isotherm fitting in this range. Contrarily, the calculation approach, which is based on the dual-site fitting of the adsorption isotherms, can describe the heat of adsorption more reliably, as the whole isotherm region can be described very well by the fit. The heats of adsorption are generally rather high with *q_st_* > 30 kJ/mol even at high loadings. At low loadings, there seem to be some indication for higher energy adsorption places (*q_st_* ~ 38–41 kJ·mol^−1^), which is supported by isotherm fitting (see below). As the materials have generally very narrow pores (which additionally enhance the heat of adsorption) [15,16] and may contain N functionalities as well as metal traces, the presence of high energy adsorption sites is clearly unsurprising. Indeed, it was shown that carbon materials with comparably narrow pores (*W* < 0.7 nm) but without pronounced chemical functionality do show values of *q_st_* = 29–30 kJ·mol^−1^ even at high loadings. The introduction of chemical functionality (as for example OH-groups) in such narrow pore size materials does however lead to increased heat of adsorption up to 35–38 kJ·mol^−1^ [15,16].

CO_2_ isotherms were fitted by a single-site and dual-site Langmuir approach (see supporting information). A single site approach could not give a good fit of the isotherms, while a dual-site fit gave reasonable results. This supports the presence of some high-energy sites. In contrast, N_2_ adsorption isotherms on PT1-600 and PT5-600, which were measured at 273.15 K (N_2_ adsorption capacity at 273.15 K and 1 bar are ~0.31 mmol·g^−1^ and ~0.29 mmol·g^−1^ for PT1-600 and PT5-600, respectively), in order to calculate the adsorption selectivity, could be fitted very well using a simple (single site) Langmuir approach. This gives additional evidence for the presence of adsorption sites favoring CO_2_ adsorption. The CO_2_/N_2_ adsorption selectivity α(CO_2_/N_2_) was calculated from the fitted isotherms using the ideal adsorbed solution theory [IAST (ideal adsorbed solution theory), see ESI for details] [28] for a gas composition of 0.15/0.85 (typical composition of flue gas, Figure 5]. We calculated also the adsorption isotherms of CO_2_ and N_2_ from the respective gas mixture at 237 K, which can be predicted on the base of IAST as well. [29,30].

Although measured at 273 K (roughly 30–50 K lower than the normal temperature of flue gas in a modern coal-fired power plant), the calculated selectivities can be used to decide whether the materials could indeed be regarded as useful CO_2_ adsorbents. IAST selectivities of ~360 and ~640 were calculated for PT1-600 and PT5-600 at 1 bar and 273.15 K. Such selectivities are high and indeed competitive when compared to other recently reported highly selective zeolite materials [30], which showed selectivity values located in the range of 100–900. With regard to the rather small pore sizes and the narrow pore size distribution, it can be speculated that the high selectivities are a consequence of kinetic sieving effects [31], but those effects may be enhanced by favorable interactions between CO_2_ and the functional carbon surface (see above). The reason for the somewhat differing selectivities of PT1-600 and PT5-600 might be related to the overall capacity of the materials (PT5-600 has a higher CO_2_ capacity at lower N_2_ capacity). To what extent finer details of the surface chemistry play a role here cannot be derived from the data so far, as both, heat of adsorption and pore size distribution, are rather similar for the most interesting materials (PT1-600 and PT5-600). The overall adsorption capacity of the materials might however require some improvement and the adsorption kinetics deserve further investigation. The uptake capacity is comparatively low to that of activated carbons [32,33] (~2.1–2.3 mmol·g^−1^ in the case of the leather skin residue derived carbons *versus* 3–6 mmol·g^−1^ in the case of activated carbons) but could probably be enhanced by activation. Usually, this leads however to a reduced selectivity as a consequence of increased pore sizes. Whether additional activation is necessary, might hence be judged on an economic basis weighing the costs of the processing step and the benefit depending on the envisaged application. Nevertheless, reported results constitute a promising basis for future and further in-depth studies.

## 3. Experimental Section

### 3.1. Materials Preparation

Leather skin residues were leftovers from processed leather to bags, wallets and related consumer products kindly donated by Serpelsa S.A. from Vic (Barcelona, Spain). The original feedstock is rabbit skin. Samples are referred to PT (for processed skin) and numbered (1, 2, 3) depending on the type of sample and treatment. PT1 and PT3 were processed leather skin cuts under a metal-free tanning natural processing (no metals added, exclusive use of tannins [34]). Comparatively, PT2 was processed and cured using titanium salts and PT4 and PT5 were processed under conventional and most extended chromium salt treatment, typical of over 80% of the leather processing industry. Different batches were also processed in this work, showing a high reproducibility in terms of textural and adsorption properties.

Carbonization of the leather residues was conducted in a microactivity reactor (P&D, Madrid, Spain) at two different temperatures (350 and 600 °C) based on results obtained from TG/DTA experiments that indicated these were optimum decomposition points of the samples. Reduced temperatures of carbonization (<250 °C) lead to materials that had very similar properties to those of the original processed leather skin. The carbonization temperature of each sample is added at the end of the label. Thus, PT1-350 is the processed skin number one carbonized at 350 °C.

**Figure 6:**
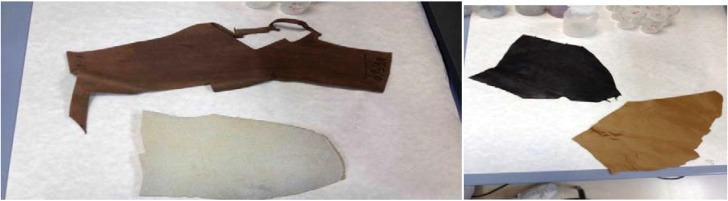
Photos of PT1 and PT5 (processed leather skin).

In a typical and simple carbonization process, 2–3 g leather skin cut in small pieces were placed into the microactivity reactor and heated up at the desired temperature (350 or 600 °C) under a flow of nitrogen (50 mL/min) at 10 °C/min and then final stabilization at the carbonization temperature for 60 min. Carbonization under oxygen or air was found to significantly mineralize the initial material, especially at higher temperatures (>350 °C). The final carbonaceous material was then obtained upon cooling down of the system and processed accordingly. Representative photos of the starting materials have been included in Figure 6.

### 3.2. Characterization of Materials

Materials were characterized by means of several techniques including nitrogen physisorption, TG/DTA, X-ray Diffraction, Diffuse Reflectance Infrared Fourier Transform Spectroscopy (DRIFTS), Transmission Electron Microscopy (TEM) and N_2_ and CO_2_ adsorption.

Thermal analysis was performed by simultaneous thermal gravimetric and differential thermal analysis (TG/DTA) measurement using a Setsys 12 Setaram thermobalance. Samples were heated in air or argon (50 mL/min) from room temperature to 900 °C at a heating rate of 10 °C·min^−1^.

XRD patterns were recorded on a Bruker AXS diffractometer with CuKα (λ = 1.5418 Å), over a 2*θ* range from 5° to 80°, using a step size of 0.01° and a counting time per step of 20 s.

Nitrogen adsorption measurements were carried out at 77.4 K using an ASAP 2010 volumetric absorption analyzer from Micromeritics. The samples were outgassed 24 h at 150 °C under vacuum (*p* < 10^−^^2^ Pa) and subsequently analyzed. The linear part of the BET (brunauer-emmett-teller theory) equation (relative pressure between 0.05 and 0.30) was used for the determination of the specific surface area. DBJH = mean pore size diameter; VBJH = pore volumes. The pore size distribution was calculated from the adsorption branch of the N_2_ physisorption isotherms and the Barret–Joyner–Halenda (BJH) formula. The cumulative pore volume VBJH was obtained from the pore size distribution (PSD) curve.

The size and morphology of carbonaceous materials were investigated using a transmission electron microscope (TEM, JEOL, Tokyo, Japan, JEM-2010HR) operated at 300 kV. Samples were suspended in ethanol and deposited straightaway on a copper grid prior to analysis.

DRIFTS experiments were conducted in a Perkin Elmer Spectrumn 100 infrared spectrometer equipped with an attenuated total reflectance (ATR) module. Attenuated total reflectance infrared (FTIR-ATR) spectra were recorded using a Perkin Elmer^®^ SpectrumTM 400 FT-IR/NIR spectrometer (Perkin Elmer Inc., Tres Cantos, Madrid, Spain) in mid-IR mode, equipped with a universal ATR (atenuated total reflectance) sampling device containing diamond/ZnSe crystal. Besides, for powdered samples an extra accessory plate with a conic awl was used which required only a few milligrams without any previous sample preparation. Spectra were acquired and then processed with the Spectrum software version 6.3.2. Spectra were scanned at room temperature in absorbance mode over the wave number range of 4000–650 cm^−1^, with a scan speed of 0.20 209 cm s^−1^ and 30 accumulations at a resolution of 4 cm^−1^. A background spectrum of air was scanned under the same instrumental conditions before each series of measurements.

CO_2_ and N_2_ adsorption/desorption isotherms at 273 and 283 K were conducted on an Autosorb-1MP instrument (Quantachrome Corp, Boynton Beach, FL, USA.). High purity gases were used and the samples were degassed prior to analysis at 150 °C and high vacuum. Initial data analysis and calculation of pore size distributions and heat of adsorption was done using the QuadraWin 5.05 software package (Quantachrome Corp). IAST calculations were based on octave and MatLab^®^ scripts, which can be accessed from the authors or via Internet at [35].

## 4. Conclusions

Leather skin residues have been converted into useful carbonaceous materials that have very promising CO_2_ sequestration properties and selected materials exhibited very high gas selectivities. Given the fact that greener useful materials can be produced from inexpensive waste even with low burn-off rates as opposed to derived from expensive monomers with time-consuming and wasteful experimental protocols [(e.g., metal-organic frameworks (MOFs)], the obtained results are rather competitive and encouraging. Additionally, materials are carbon-based, which makes them less sensitive to moisture, which is an important fact to keep in mind for CO_2_ adsorption. Although the materials might not be relevant to applications in power plants (which require large amount of adsorbents), they may find other interesting CO_2_-related markets (e.g., air supply in aircrafts).

## References

[1] Constanza R., Daly H.E. (1992). Natural capital and sustainable development. Conserv. Biol..

[2] Lin C.S.K., Pfaltzgraff L., Herrero-Davila L., Mubofu E.B., Abderrahim S., Clark J.H., Koutinas A., Kopsahelis N., Stamatelou K., Dickson F. (2012). Food waste as a valuable resource for the production of chemicals, materials and fuels. Current situation and global perspective. Energy Environ. Sci..

[3] Cabeza L., Taylo M.M., Di Maio G.L., Brown E., Marmer W.N., Carrió R., Celma J., Cot J. (1998). Processing of leather waste: pilot scale studies on chrome shavings. Isolation of potentially valuable protein products and chromium. Waste Manag..

[4] Rao J.R., Thanikaivelan P., Sreeram K.J., Nair B.U. (2002). Green route for the disposal of chrome shavings (chromium containing solid waste) in tanning industry. Environ. Sci. Technol..

[5] United Nations Industrial Development Organization (UNIDO) Wastes Generated in the Leather Products Industry. http://www.unido.org/fileadmin/import/userfiles/timminsk/leatherpanel14ctcwastes.pdf.

[6] Catalina M., Antunes A.P.M., Attenburrow G.E., Cot J., Covington A.D., Phillips P.S. (2007). Sustainable management of waste-reduction of the chromium content of tannery solid waste as a step in the cleaner production of gelatin. J. Solid Waste Technol. Manag..

[7] Catalina M., Attenburrow G.E, Cot J., Covington A.D., Antunes A.P.M. (2011). Influence of crosslinkers and crosslinking method on the properties of gelatin films extracted from leather solid waste. J. Appl. Polym. Sci..

[8] Catalina M., Cot J., Balu A.M., Serrano-Ruiz J.C., Luque R. (2012). Tailor-made biopolymers from leather waste valorisation. Green Chem..

[9] Ashokkumar M., Narayanan N.T., Reddy A.L.M., Gupta B.K., Chandrasekaran B., Talapatra S., Ajayan P.M., Thanikaivelan P. (2012). Transforming collagen wastes into doped nanocarbons for sustainable energy applications. Green Chem..

[10] D’Alessandro D.M., Smit B., Long J.R. (2010). Carbon dioxide capture: Prospects for new materials. Angew. Chem. Int. Ed..

[11] Lu W., Sculley J.P., Yuan D., Krishna R., Wei Z., Zhou H.C. (2012). Polyamine-tethered porous polymer networks for carbon dioxide capture from flue gas. Angew. Chem. Int. Ed..

[12] McDonald T.M., Lee W.R., Mason J.A., Wiers B.M., Hong C.S., Long J.R. (2012). Capture of carbon dioxide from air and flue gas in the alkylamine-appended metal-organic framework mmen-Mg_2_(dobpdc). J. Am. Chem.Soc..

[13] Rodriguez-Reinoso F., Lopez-Gonzalez J.D., Berenguer C. (1982). Activated carbons from almond shells—I: Preparation and characterization by nitrogen adsorption. Carbon.

[14] Lozano-Castelló D., Cazorla-Amoros D., Linares-Solano A. (2004). Usefulness of CO_2_ adsorption at 273 K for the characterization of porous carbons. Carbon.

[15] Yu L., Falco C., Weber J., White R., Howe J.Y., Titirici M.M. (2012). Carbohydrate-derived hydrothermal carbons: A thorough characterization study. Langmuir.

[16] Baccile N., Weber J., Falco C., Titirici M.M., Titirici M.M. (2013). Sustainable Carbon Materials from Hydrothermal Processes.

[17] Ritter N., Senkovska I., Kaskel S., Weber J. (2011). Intrinsically microporous poly(imide)s: Structure-porosity relationship studied by gas sorption and X-ray scattering. Macromolecules.

[18] Budarin V., Clark J.H., Hardy J.J.E., Luque R., Macquarrie D.J., Milkowski K., Tavener S.J., Wilson A.J. (2006). Starbons: New starch-derived mesoporous carbonaceous materials with tunable properties. Angew. Chem. Int. Ed..

[19] Xing X., Liu C., Zhou Z., Zhang L., Zhou J., Zhou S., Yan Z., Gao H., Wang G., Qiao S.Z. (2012). Superior CO_2_ uptake of N-doped activated carbon through hydrogen-bonding interaction. Energy Environ. Sci..

[20] Maroto-Valer M.M., Tang Z., Zhang Y. (2005). CO_2_ capture by activated and impregnated anthracites. Fuel Process. Technol..

[21] Grondein A., Belanger D. (2011). Chemical modification of carbon powders with aminophenyl and aryl-aliphatic amine groups by reduction of in situ generated diazonium cations: Applicability of the grafted powder towards CO_2_ capture. Fuel.

[22] Li Q., Zhang J., Feng D., Wu Z., Wu Q., Park S.S., Ha C., Zhao D. (2010). Facile synthesis of porous carbon nitride spheres with hierarchical three-dimensional mesostructures for CO_2_ capture. Nano Res..

[23] Sevilla M., Valle-Vigón P., Fuertes A.B. (2011). N-doped polypyrrole-based porous carbons for CO_2_ capture. Adv. Funct. Mater..

[24] Gutierrez M.C., Carriazo D., Ania C.O., Parra J.B., Ferrer M.L., Monte F.D. (2011). Deep eutectic solvents as both precursors and structure directing agents in the synthesis of nitrogen doped hiearchical carbons highly suitable for CO_2_ capture. Energy Environ. Sci..

[25] Hao G., Li W., Qian D., Lu A. (2010). Rapid synthesis of nitrogen-doped porous carbon monolith for CO_2_ capture. Adv. Mater..

[26] Sevilla M., Parra J.B., Fuertes A.B. (2013). Assessment of the role of micropore size and N-doping in CO_2_ capture by porous carbons. ACS Appl. Mater. Interfaces.

[27] Vishnyakov A., Ravikovitch P.I., Neimark A.V. (1999). Molecular level models for CO_2_ sorption in nanopores. Langmuir.

[28] Mason J.A., Sumida K., Herm Z.R., Krishna R., Long J.R. (2011). Evaluating metal-organic framworks for post-combustion carbon dioxidecapture via temperature swing adsorption. Energy Environ. Sci..

[29] Myers A.L., Prausnitz J.M. (1965). Thermodynamics of mixed-gas adsorption. AIChE J..

[30] Akhtar F., Liu Q., Hedin N., Bergström L. (2012). Strong and binder free structured zeolite sorbents with very high CO_2_-over-N_2_ selectivities and high capacities to adsorb CO_2_ rapidly. Energy Environ. Sci..

[31] Hedin N., Andersson L., Bergström L., Yan J. (2013). Adsorbents for the post-combustion capture of CO_2_ using rapid temperature swing or vacuum swing adsorption. Appl. Energy.

[32] Sevilla M., Fuertes A.B. (2011). Sustainable porous carbons with a superior performance for CO_2_ capture. Energy Environ. Sci..

[33] Sevilla M., Fuertes A.B. (2012). CO_2_ adsorption by activated templated carbons. J. Colloid Interface Sci..

[34] SERPELSA Furs SA Homepage. www.serpelsa.com.

[35] Max Plank Institute of Colloids and Interfaces, Groups of Porous Polymers. http://www.mpikg.mpg.de/144866/xDownloads.

